# BAX-mediated ammonia-driven cell death: a novel prognostic and therapeutic target in clear cell renal cell carcinoma

**DOI:** 10.1186/s40246-025-00764-3

**Published:** 2025-05-17

**Authors:** Xi Zhang, Zijie Yu, Lu Yin, Qiang Li, Shaohua He, Heng Li, Jian Li, Lian Sheng, Hongfei Wu, Hongqi Chen, Xiaoxu Zhu, Yang Lv

**Affiliations:** 1 Department of Urology, The Affiliated Jiangsu Shengze Hospital of Nanjing Medical University, Suzhou, 215000 People’s Republic of China; 2 Department of Urology, The First Affiliated Hospital of Nanjing Medical University, Nanjing, 210029 People’s Republic of China; 3 Yunnan Key Laboratory of Dai and Yi Medicines, Yunnan University of Chinese Medicine, Kunming, Yunnan, 650500 People’s Republic of China; 4 Suzhou Industrial Park Xietang Community Health Service Center, Suzhou, 215000 People’s Republic of China; 5 Suzhou Wujiang District Hospital of Traditional Chinese Medicine (Suzhou Wujiang District Second People’s Hospital), Suzhou, 215000 People’s Republic of China

**Keywords:** Clear cell renal cell carcinoma, Ammonia-related cell death, Prognostic characteristics, BAX, Single-cell RNA sequencing

## Abstract

**Background:**

ccRCC (clear cell renal cell carcinoma) is characterized by metabolic reprogramming and immunosuppression, leading to poor clinical prognosis. In recent years, ammonia-related cell death has attracted increasing attention as a novel mechanism related to tumor progression, but its role in ccRCC has not been clarified.

**Methods:**

In this study, the Ammonia-related Signature (AS) of ccRCC was constructed by integrating bioinformatics analysis and experimental verification. Multiple independent cohorts including TCGA, PMID 35,440,542 cohort, E-MTAB-1980, and GSE29609 were used to evaluate prognostic accuracy and clinical relevance, and biological functions of key ammonia related genes were explored by cell proliferation, clonal formation, migration, and invasion assays. ScRNA-seq was used to analyze interaction between AS and immune cells in ccRCC.

**Results:**

The ammonia-related prognostic model demonstrated robust predictive power in multiple datasets. The high AS group of patients with poor prognosis, and the tumor mutation load, immunosuppressive cell infiltration level and immune checkpoint molecular expression were higher. BAX was a key ammonia-related gene closely related to tumor progression, and its knockdown obviously inhibit proliferation, migration and invasion of ccRCC cells. Single-cell analysis confirmed the activation of ammonia-related signaling pathways in the tumor microenvironment, in particular revealing specific interactions between BAX-positive tumor cells and immunosuppressive cell populations.

**Conclusion:**

The ammonia-related cell death pathway, especially BAX, can be employed as a potential prognostic marker and therapeutic target for ccRCC, providing new ideas for individualized treatment strategies to overcome immunosuppression and improve clinical prognosis.

**Supplementary Information:**

The online version contains supplementary material available at 10.1186/s40246-025-00764-3.

## Introduction

Clear cell renal cell carcinoma (ccRCC) is the most common subtype of kidney cancer, with clinical features such as strong aggressiveness and increasing global incidence [1, 2]. Although recent advances in therapeutic strategies (including surgical resection, targeted therapy, and immunotherapy), patient prognosis remains unsatisfactory due to high tumor heterogeneity, intrinsic treatment resistance, and lack of effective predictive markers [3]. Therefore, the exploration of novel and reliable prognostic markers is essential for individualized clinical management and improved outcomes.

Regulated cell death (RCD), including apoptosis, necrotic apoptosis, iron death, cuproptosis, copper death and other classical and emerging forms, plays an important role in maintaining cell homeostasis and regulating tumor biological behavior [4–6]. Dysregulation of these cell death pathways not only participates in tumor occurrence and development, but also can reshape tumor immune microenvironment and affect treatment response and patient prognosis [7–9]. Therefore, targeting specific cell death mechanisms provides new ideas for cancer treatment.

Recent studies have found that ammonia-related cell death, as a new type of RCD, is characterized by abnormal ammonia metabolism, mitochondrial dysfunction, oxidative stress and cell homeostasis imbalance [10, 11]. Elevated ammonia levels can induce cell stress and eventually lead to cell death through mechanisms such as triggering mitochondrial permeability conversion and increasing reactive oxygen species (ROS) production [12, 13]. In addition, ammonia metabolism disorder is closely related to tumor metabolic reprogramming, immune escape and aggressive phenotype [14]. However, the role of ammonia-related cell death pathway in the progression and prognosis of ccRCC remains unclear, and further studies are needed.

In this study, a multi-group bioinformatics method was used to systematically screen ammonia-related genes and construct a prognostic model of ccRCC to verify its cross-cohort prediction efficiency. High-risk patients present with adverse clinical outcomes, genomic instability, high expression of immune checkpoint molecules, and immunosuppressive microenvironment characteristics. BAX was identified as the key regulatory factor. These findings suggest that the ammonia-associated cell death pathway, especially BAX, could be a prognostic marker and a potential intervention target for individualized therapy of ccRCC.

## Methods

### Data acquisition and processing

ccRCC gene expression profiles, somatic cell mutation data, and clinical information are derived from the following public databases: TCGA (The Cancer Genome Atlas), GSE29609 cohort [14], E-MTAB-1980, PMID 35,440,542 cohort, and IMvigor210 datasets. scRNA-seq data were obtained from GSE131685 [15] and GSE171306 [16]. All raw data was normalized by log2 transformation and batch effects were corrected using ComBat algorithms. Based on the MSigDB and KEGG gene sets related to ammonia metabolism, Combined with literature search [10], candidate genes related to ammonia metabolism and cell death were systematically screened.

### Construction and validation of prognostic model

Through systematic evaluation of multiple machine learning algorithms, the optimal prognosis prediction model of ccRCC was screened. The concordance index (C-index) was employed to quantify prognostic model to predict performance, and cross-cohort validation was performed on the TCGA-KIRC cohort, GSE29609 validation set, E-MTAB-1980 cohort, and independent data sets from PMID 35,440,542 cohort. Finally, the algorithm framework with the highest C-index was selected and the Cox proportional risk model was used to build an Ammonia-related Signature (AS) system. The consistency of the model’s predicted survival probability with actual observed values was evaluated by drawing calibration curves, and the net benefit rate of the model in clinical decision making was validated by using decision curve analysis (DCA).

### Immune landscape and immunotherapy response analysis

The ssGSEA (Single-sample Gene Set Enrichment Analysis) algorithm was used to evaluate the infiltration proportion of 22 immune cell subsets in tumor tissues, integrate the expression profile of immune checkpoint molecules and the classification of immune subtypes (C1-C6), and systematically analyze the immune microenvironment characteristics of ccRCC. Based on somatic mutation data from TCGA database, the number of non-synonymous single nucleotide variation (SNV) was calculated to quantify tumor mutation burden (TMB). The efficacy of AS in predicting objective response rate (ORR) of immunotherapy was further validated using an IMvigor210 cohort treated with anti-PD-L1. Tumor Immunophenotype Tracking (TIP) algorithm was used to dynamically analyze the changes in the functional status of immune cells before and after treatment. Detailed parameters of the experimental method are shown in supplementary Table 1.

## Results

### Multi-method screening and cross-cohort validation of the ammonia-related cell death prognostic model

**Fig. 1:**
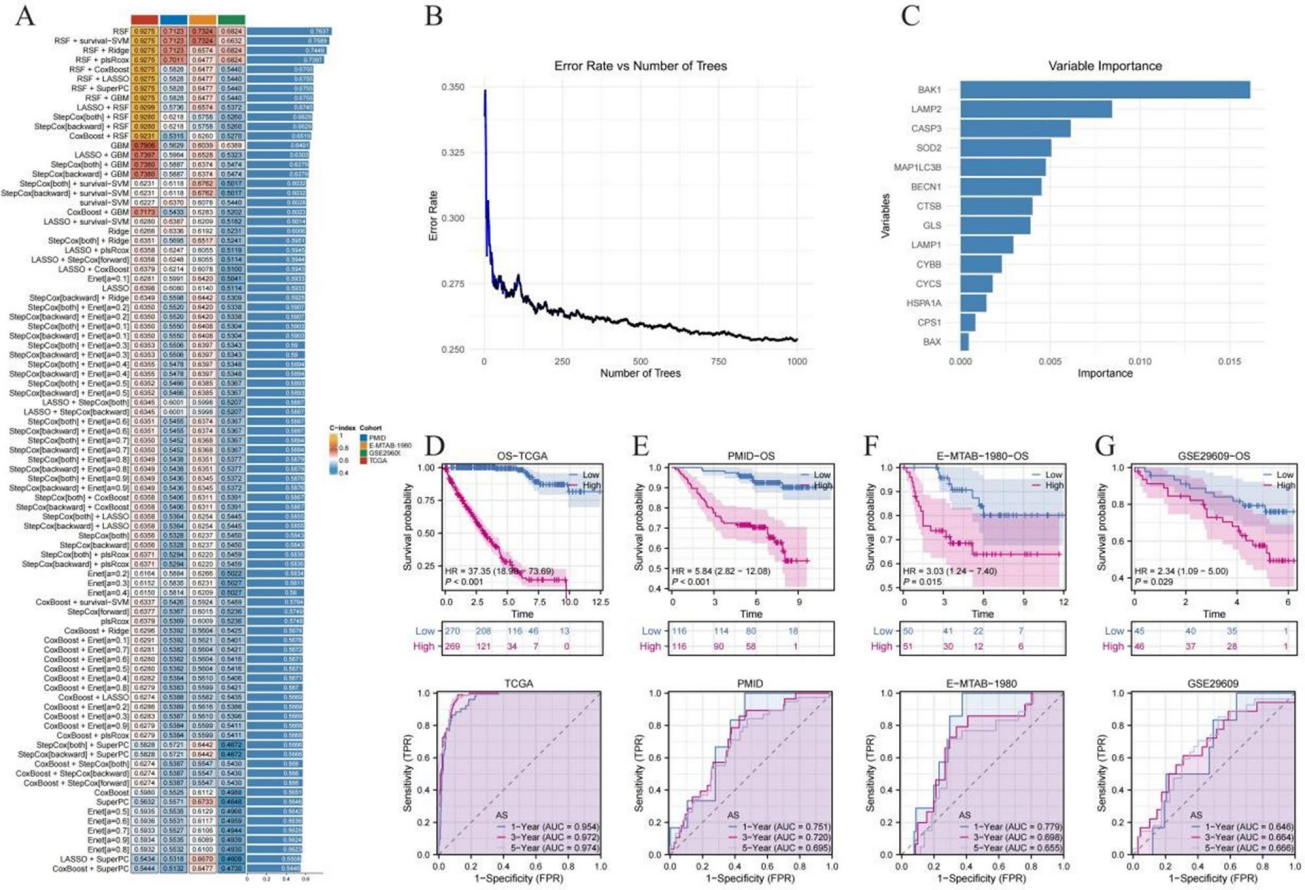
Construction, validation, and performance assessment of the ammonia death-related prognostic model. (**A**) Bar chart compares the C-index of different combinations of Cox regression machine learning algorithms across multiple validation queues. (**B**) The error rate curve shows the convergence of the random survival forest model as the decision tree increases. (**C**) Bar chart showing the relative importance of ammonia metabolism-related genes in the prognostic model. (**D**-**G**) KM (Kaplan-Meier) survival curve and time-dependent ROC (Receiver Operating Characteristic) curve to assess the effectiveness of risk stratification in independent cohorts

In this study, four independent cohorts (TCGA-KIRC, PMID 35440542 [17], E-MTAB-1980, and GSE29609) were integrated, and multiple machine learning algorithms in combination with Cox regression were employed to construct and screen a prognostic model based on ammonia-related cell death genes (ARGs). The predictive performance of each model was evaluated using C-index (Concordance Index). We compared LASSO, Elastic Net, and Random Survival Forest (RSF) models. While LASSO and Elastic Net perform linear modeling with variable selection, RSF is a non-parametric method that captures non-linear interactions and does not rely on the proportional hazards assumption. RSF consistently showed the highest C-index across validation cohorts and was thus selected as the final model (Fig. 1A). To assess the stability of the RSF approach, the prediction error rate was observed to plateau as the number of decision trees increased, suggesting favorable generalization performance once a sufficiently large number of trees were included (Fig. 1B). According to the variable importance rankings generated by the RSF model, genes such as BAK1, LAMP2, CASP3, SOD2, and MAP1LC3B made substantial contributions, implying their crucial roles in ammonia-related regulation and prognostic prediction (Fig. 1C). Furthermore, patients were stratified into high- and low-risk groups according to the scores derived from the model, and KM (Kaplan-Meier) analysis in all four cohorts consistently revealed significantly poorer outcomes in the high-risk group. The time-dependent ROC (Receiver Operating Characteristic) analysis demonstrated relatively high AUC (Area Under the Curve) values for 1-, 3-, and 5-year survival predictions, confirming the stability and reproducibility of this model across multiple cohorts and providing robust support for individualized risk assessment in ccRCC patients (Fig. 1D-G).

### Prognostic characteristic of the ammonia-related cell death prognostic model

**Fig. 2:**
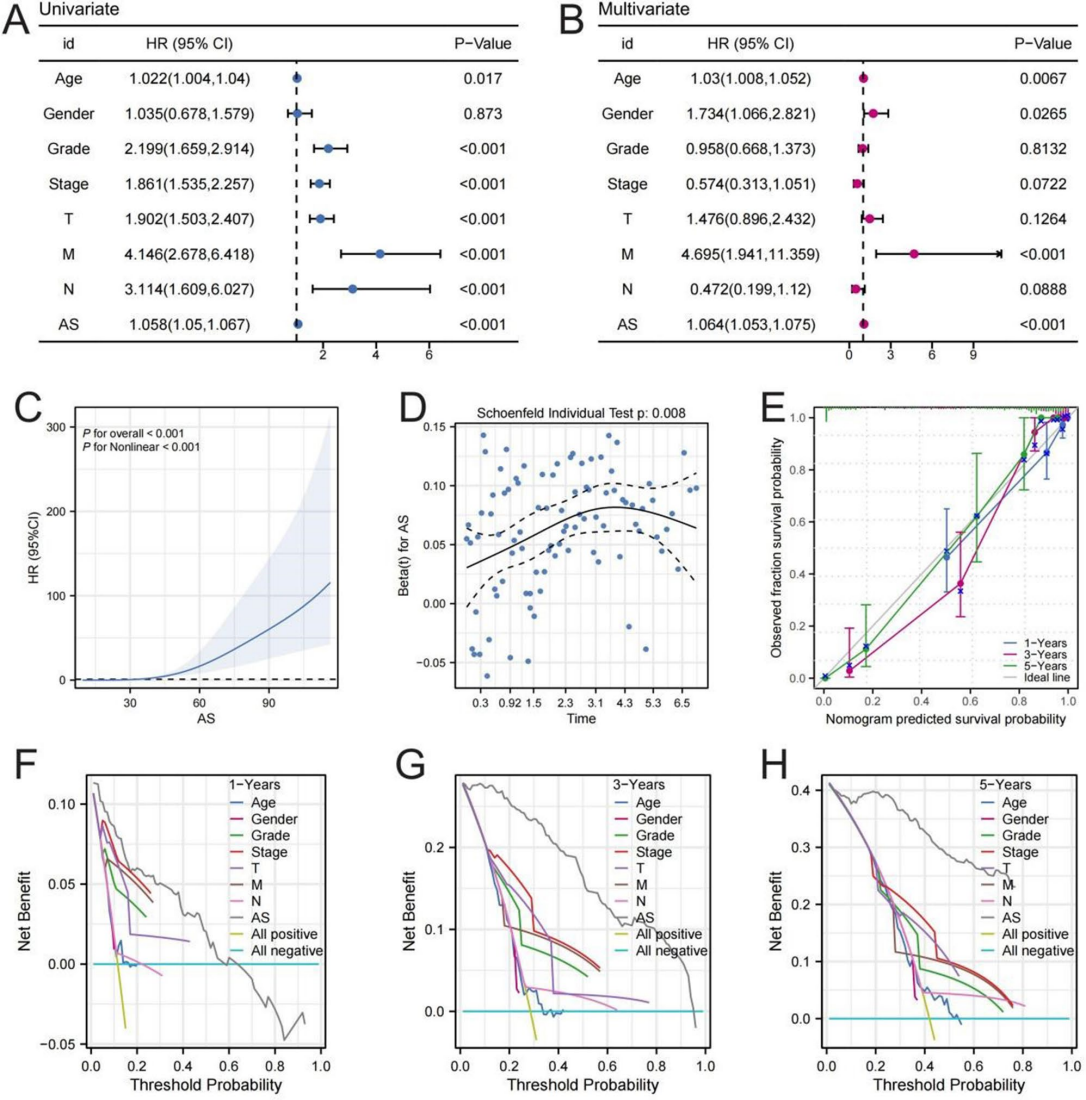
Prognostic characteristic of the ammonia death-related prognostic model. (**A**, **B**) Forest maps showing univariate/multivariate Cox regression analysis of clinical and molecular variables. (**C**) Restricted Cubic spline analysis revealed a non-linear association between AS and total survival risk. (**D**) Schoenfeld residual test to verify the proportional risk hypothesis. (**E**) Calibration curves evaluate the prediction of 1/3/5 year survival probability - observational consistency. (**F**-**H**) Decision Curve Analysis evaluated the clinical net benefit of the model at different risk thresholds

Univariate and multivariate Cox regression analyses were used to evaluate prognostic effects of clinical parameters and AS on overall survival (OS). The results showed that AS was an independent prognostic factor, confirming its independent predictive value for ccRCC outcome (Fig. 2A-B). Restricted cubic spline analysis showed a significant positive association between AS levels and the mortality hazard ratio (HR), suggesting a non-linear association with patient outcomes (Fig. 2C). Significant deviations were found when Schoenfeld residuals were used to evaluate the proportional risk hypothesis, suggesting that AS may have a time-dependent effect in the Cox model or need to be stratified (Fig. 2D). Calibration curves of the nomogram show that the predicted survival rates at 1 -, 3 -, and 5-year time points are highly consistent with actual observations, confirming the prediction accuracy of the model (Fig. 2E). Decision curve analysis showed that the predictive model incorporating AS had a higher net benefit rate at 1, 3, and 5 years than the traditional strategy (“ all positive “or” all negative “), highlighting the clinical value of AS over a single clinical variable (Fig. 2F).

### Differences between immune cell infiltration and tumor microenvironment in ammonia-related cell death prognostic models

**Fig. 3:**
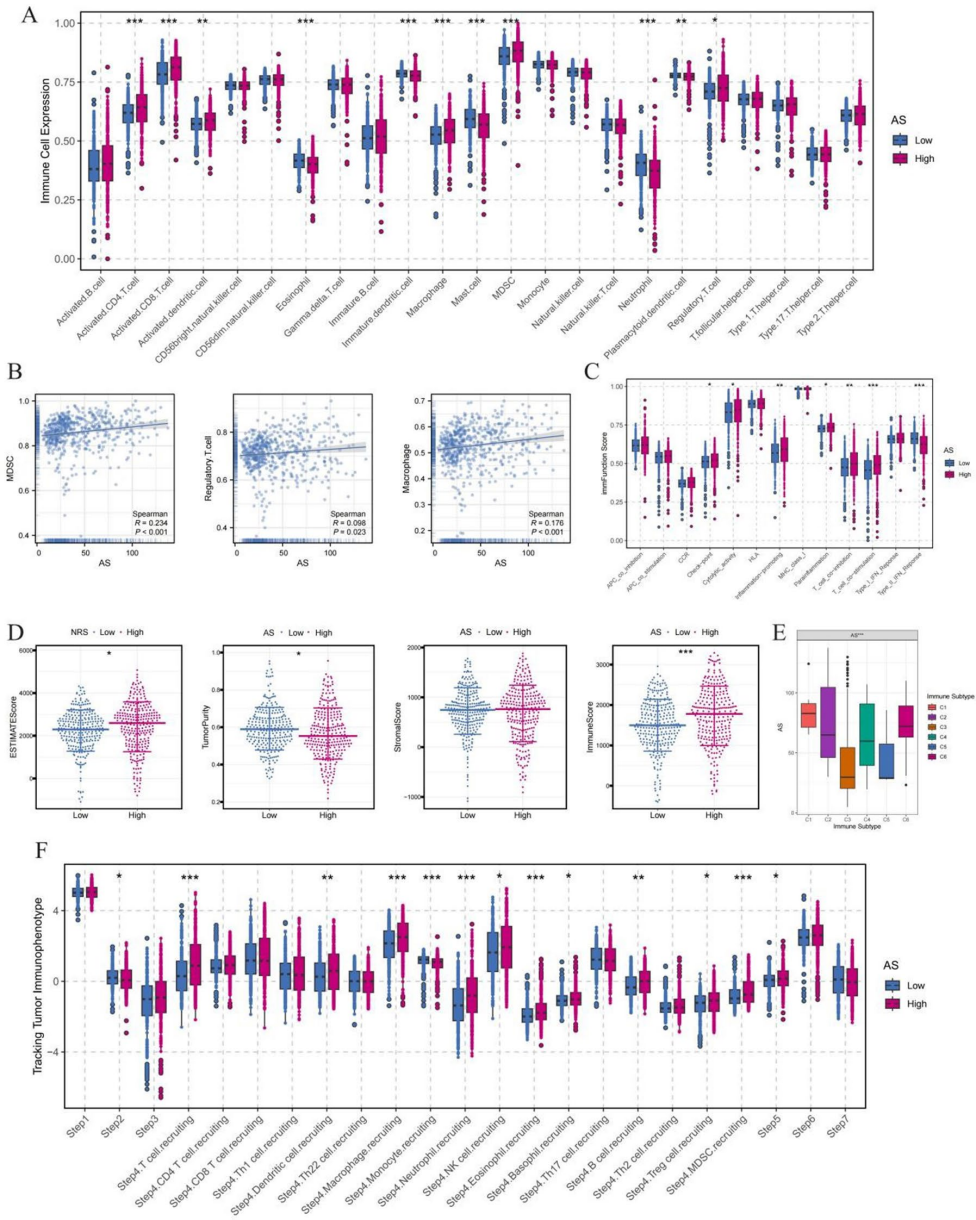
Immune infiltration and tumor microenvironment analysis of the ammonia death-related prognostic model. (**A**) Box plots compared the difference in immune cell infiltration levels between the low/high AS groups. (**B**) Scatter plots showed the correlation between AS (Ammonia-related Signature) and immunosuppressive cells. (**C**) Boxplot was used to analyze the differences in immune-related functional activity scores between AS groups. (**D**) The violin plot presents the TME (Tumor Microenvironment) score distribution for AS stratification. (**E**) Distribution characteristics of immune subtypes (C1-C6) in low/high AS tumors. (**F**) Box plots showing differences in immunosuppression between AS groups revealed by TIP parameters

There were significant differences in immune cell infiltration between the low AS group and the high AS group, and the levels of multiple immune cell subsets were significantly increased in the high AS group (Fig. 3A). Further correlation analysis confirmed that AS score was significantly positively correlated with key immunosuppressive cells, suggesting that the rise of AS may promote the formation of immunosuppressive microenvironment (Fig. 3B). Functional analysis of immune-related features showed that the expression of immunosuppressive pathways and checkpoint molecules was significantly up-regulated in the high-AS group, indicating enhanced immune escape ability ((Fig. 3C). Tumor microenvironment (TME) analysis showed that the ESTIMATE score, immune score, and stromal score were significantly increased in the high AS group, while the tumor purity decreased, reflecting increased infiltration of immune and stromal components, a feature often associated with tumor progression and immune escape (Fig. 3D). The classification of immune subtypes further revealed that the high-AS group was significantly enriched in the C6 subtype with strong immunosuppressive properties and poor clinical prognosis (Fig. 3E). Consistent with this, the degree of infiltration of immunosuppressive phenotypes, such as regulatory T cells (Tregs) and MDSCs (Myeloid-derived suppressor cells), was significantly increased in high-AS tumors (Fig. 3F). In conclusion, AS score is closely related to immunosuppressive TME, which may be a key mechanism of immune escape and poor prognosis in patients with high AS ccRCC.

### Genomic alterations and immunotherapy response of the ammonia-related cell death prognostic model

**Fig. 4:**
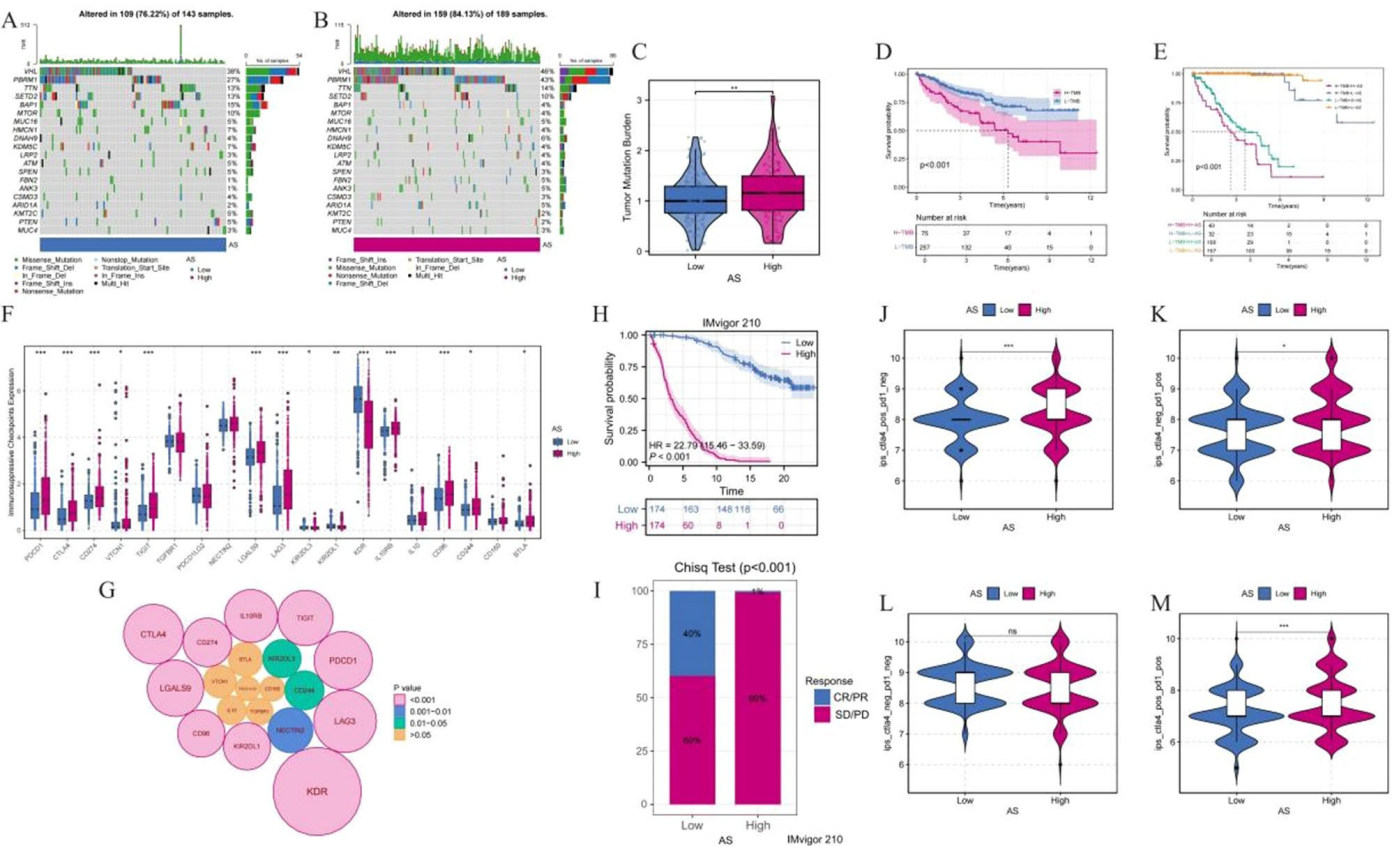
Genomic alterations, immune checkpoint expression, and immunotherapy outcomes of the ammonia death-related prognostic model. (**A**, **B**) The mutant profiles were different from those of low/high AS tumors. (**C**) The distribution of TMB (Tumor Mutation Burden) among AS groups was analyzed by violin plot. (D, E) KM survival analysis of TMB stratified patients. (**F**) Boxplot showing the differential expression of immune checkpoint genes between groups. (**G**) Bubble maps quantified the correlation between key immune checkpoint molecules and AS. (**H**) AS stratified immunotherapy survival analysis in the IMvigor210 cohort. (**I**) Bar charts compared objective response rates (CR/PR vs. SD/PD) among subgroups of AS. (**J**-**M**) Violin plot shows the expression pattern of immunotherapy-related biomarkers in patients with low/high AS

Compared with the low AS group, the high AS group showed a higher mutation frequency in key genes such as PBRM1, SETD2, and BAP1 (Fig. 4A-B). TMB was significantly increased in the high AS group (Fig. 4C), suggesting increased genomic instability. Survival analysis showed that OS was significantly shortened in patients with high TMB (Fig. 4D), and joint stratified analysis showed that patients with high AS combined with high TMB had the worst prognosis (Fig. 4E). Molecular evaluation of immune checkpoint showed that the expression of immunosuppressive checkpoints in the high AS group was significantly up-regulated (Fig. 4F). Correlation network analysis provided additional evidence for significant enrichment of immunosuppressive molecules in high-AS tumors (Fig. 4G). A validated study based on the IMvigor210 cohort showed that immunotherapy outcomes in patients with high AS were significantly worse than those in the low AS group (Fig. 4H), with a complete/partial response (CR/PR) rate of only 1% compared to 40% in the low AS group (Fig. 4I). IPS analysis showed a significant increase in IPS values in patients with high AS treated with CTLA4 ± PD1 inhibitors, indicating a greater sensitivity to PD-1/CTLA-4 blocking therapy. These results further confirm the correlation between high AS levels and immunosuppressive tumor microenvironment and treatment resistance. In summary, high-AS tumors have the following typical characteristics: elevated tumor mutation load, enhanced activation of immunosuppressive signaling pathways, and poor clinical response to immune checkpoint blocking therapy. These findings suggest that the AS score may serve as a potential biomarker for predicting immunotherapy resistance in ccRCC patients.

### Single-cell profiling of ammonia-related signatures and immunosuppressive cell interactions in CcRCC

**Fig. 5:**
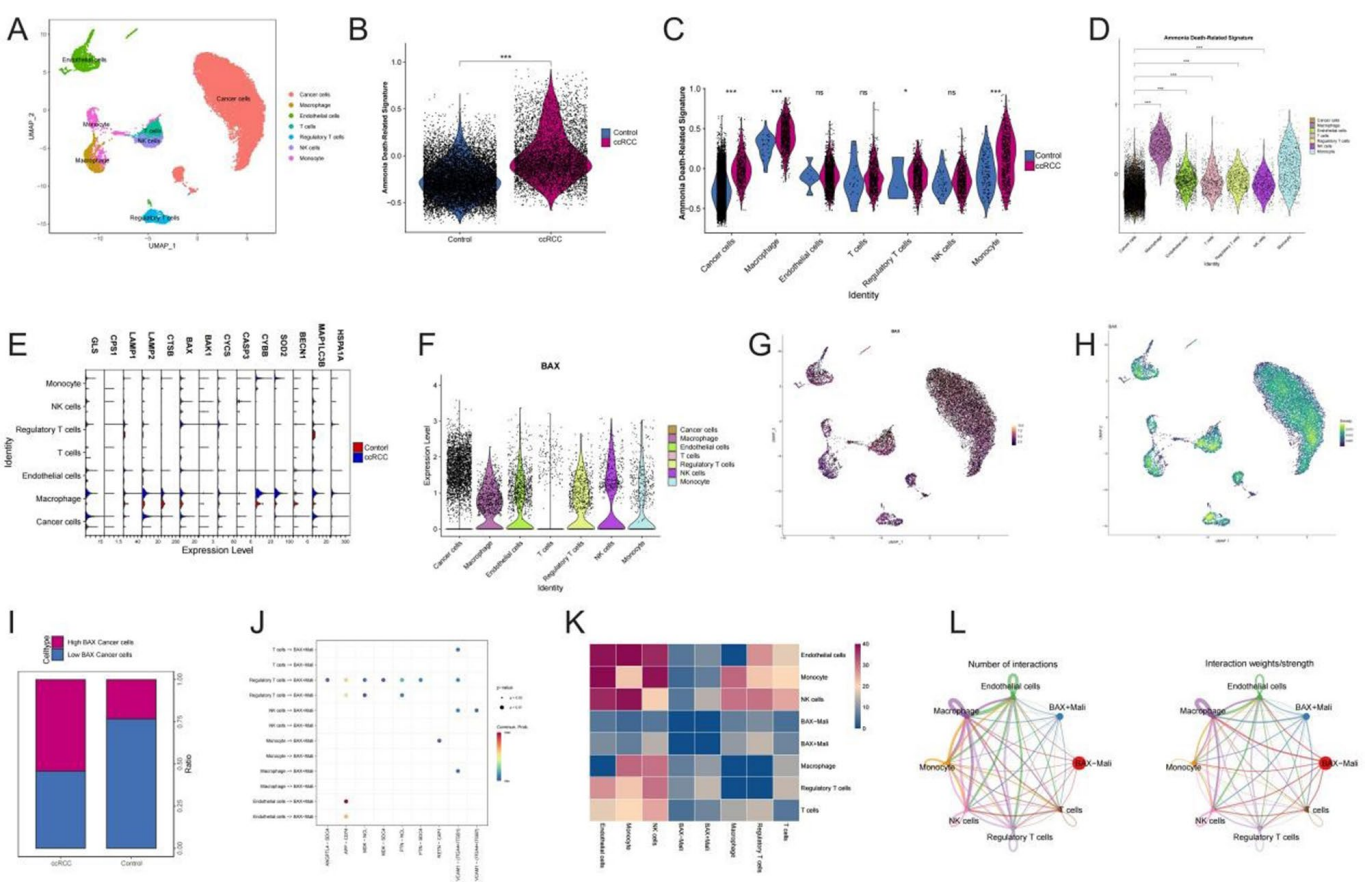
Single-cell characterization of ammonia death-related signatures in ccrcc. (**A**) UMAP (Uniform Manifold Approximation and Projection) mapping of major cell populations. (**B**) Violin chart compared AS of normal and ccRCC cells. (**C**, **D**) Heat maps showing the cell type-specific expression of ammonia metabolization-related genes in immune/stromal subsets. (**E**) The dot plots showed the differential expression of genes related to ammonia metabolism in each cell population. (**F**) Violin plot showed the distribution of BAX expression in different cell types. (**G**, **H**) Spatial UMAP projection map BAX representation localization in TME. (**I**) Stacked bar plots quantified the proportion of tumor cells with high/low BAX expression. (**J**) Bubble map analysis of ligand-receptor interactions between BAX + tumor cells and immunosuppressed populations. (**K**) Heat maps showing the correlation between ammonia metabolism-related cell clusters and BAX-associated immune subsets. (**L**) Network diagram summarizes the frequency and intensity of interactions between BAX + tumor cells and immunosuppressive components

The cell heterogeneity of ccRCC was systematically analyzed by scRNA-seq combined with UMAP (Uniform Manifold Approximation and Projection) visualization technology. Major cell subsets such as malignant cells, endothelial cells, macrophages, monocytes, natural killer (NK) cells, Tregs, and conventional T cells were clearly identified (Fig. 5A). Comparative analysis showed that ammonium-related characteristic genes in ccRCC tumor cells were significantly up-regulated, and were specifically enriched in malignant cells, macrophages, and vascular endothelial cells, suggesting a key role in tumor immune regulation and progression (Fig. 5B-D). The violin plot reveals a cell-type-specific pattern of ammonia-related gene expression, in which malignant cells and tumor-associated macrophages exhibit significant activation characteristics (Fig. 5E). It is worth noting that BAX gene showed high expression specific to tumor cells and showed obvious spatial localization characteristics in cancer nests (Fig. 5F-H), and the proportion of malignant cells with high expression of BAX in ccRCC samples significantly increased (Fig. 5I). Cell communication analysis revealed an enhanced ligand-receptor interaction network between BAX + tumor cells and immunosuppressive components such as macrophages, monocytes and Tregs, especially involving immune regulatory pathways such as TGF-β and PD-1/PD-L1 (Fig. 5J-L). These findings suggest that ammonia-related molecular interactions may drive the formation of immunosuppressive microenvironment, promote immune escape of ccRCC and tumor progression.

### Differential expression and prognostic significance of ammonia-related genes in CcRCC

**Fig. 6:**
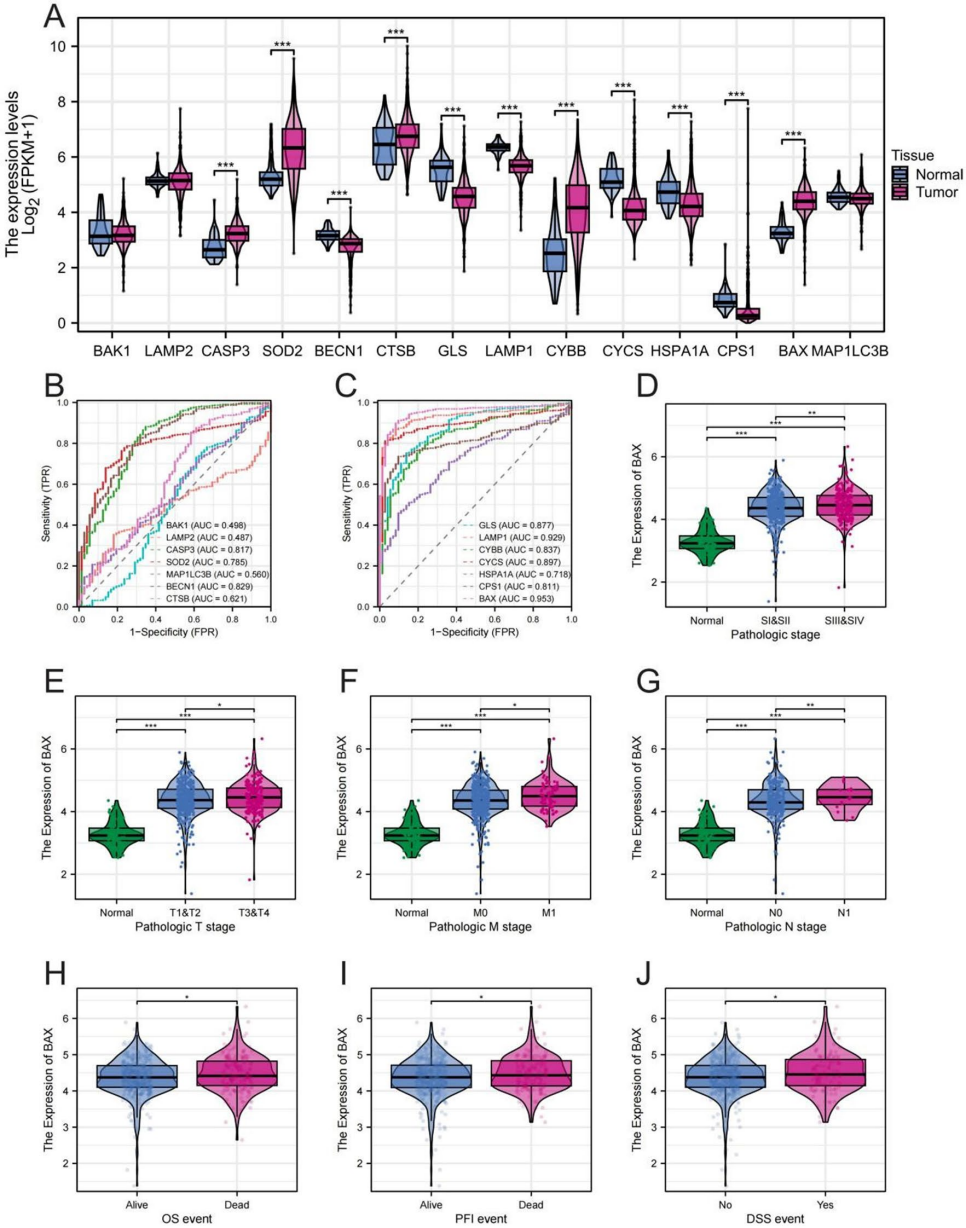
Differential expression and clinical relevance of ammonia death-related genes (**A**) Violin plot shows the difference in expression of key ammonia metabolism-related genes in normal tissues and ccRCC. (**B**, **C**) ROC (Receiver Operating Characteristic) curves evaluated the diagnostic efficacy of these genes and labeled the AUC. (**D**-**G**) Box plot was used to analyze the expression level of BAX in different pathological grades. (**H**-**J**) Comparative boxplot was used to evaluate the association of BAX expression with OS, progression-free survival and disease-specific survival

Transcriptome analysis showed that the expression of ARGs was significantly disordered in ccRCC, and most of the genes were significantly differentially expressed between tumors and adjacent normal tissues (Fig. 6A). ROC curve analysis showed that the screened ARGs had excellent diagnostic efficacy, with BAX displaying the highest AUC value in distinguishing benign and malignant tissues (Fig. 6B-C). It is worth noting that, as a promising biomarker, the expression level of BAX showed stage-dependent up-modulation and was positively correlated with advanced clinical stages (Fig. 6D-G). More importantly, high BAX expression was significantly associated with adverse clinical outcomes, including reduced OS (Fig. 6J), reduced progression-free intervals, and decreased disease-specific survival. Together, these findings establish ARGs, particularly BAX, as a key molecular determinant of tumor progression and poor prognosis in ccRCC.

## Pan-cancer analysis of BAX

To further explore the pan-cancer relevance of BAX, we analyzed its expression patterns and prognostic significance across 33 tumor types from TCGA. Figure S1A demonstrated that BAX expression was significantly upregulated in multiple cancer types, including KIRC, LIHC, CHOL, COAD, GBM, and PAAD, compared to matched normal tissues (*P* < 0.001). Conversely, downregulation was observed in cancers such as THCA and PRAD. Figure S1B–D displayed the prognostic value of BAX in OS, disease-specific survival (DSS), and progression-free interval (PFI), respectively. In several tumors, including KIRC, LIHC, LGG, and PAAD, higher BAX expression was significantly associated with worse prognosis (*P* < 0.05), suggesting a potential tumor-promoting function in specific cancer contexts. These findings further supported the context-dependent role of BAX in tumor progression and validate its potential as a pan-cancer prognostic biomarker.

### BAX knockdown inhibits proliferation, colony formation, migration, and invasion in CcRCC

**Fig. 7:**
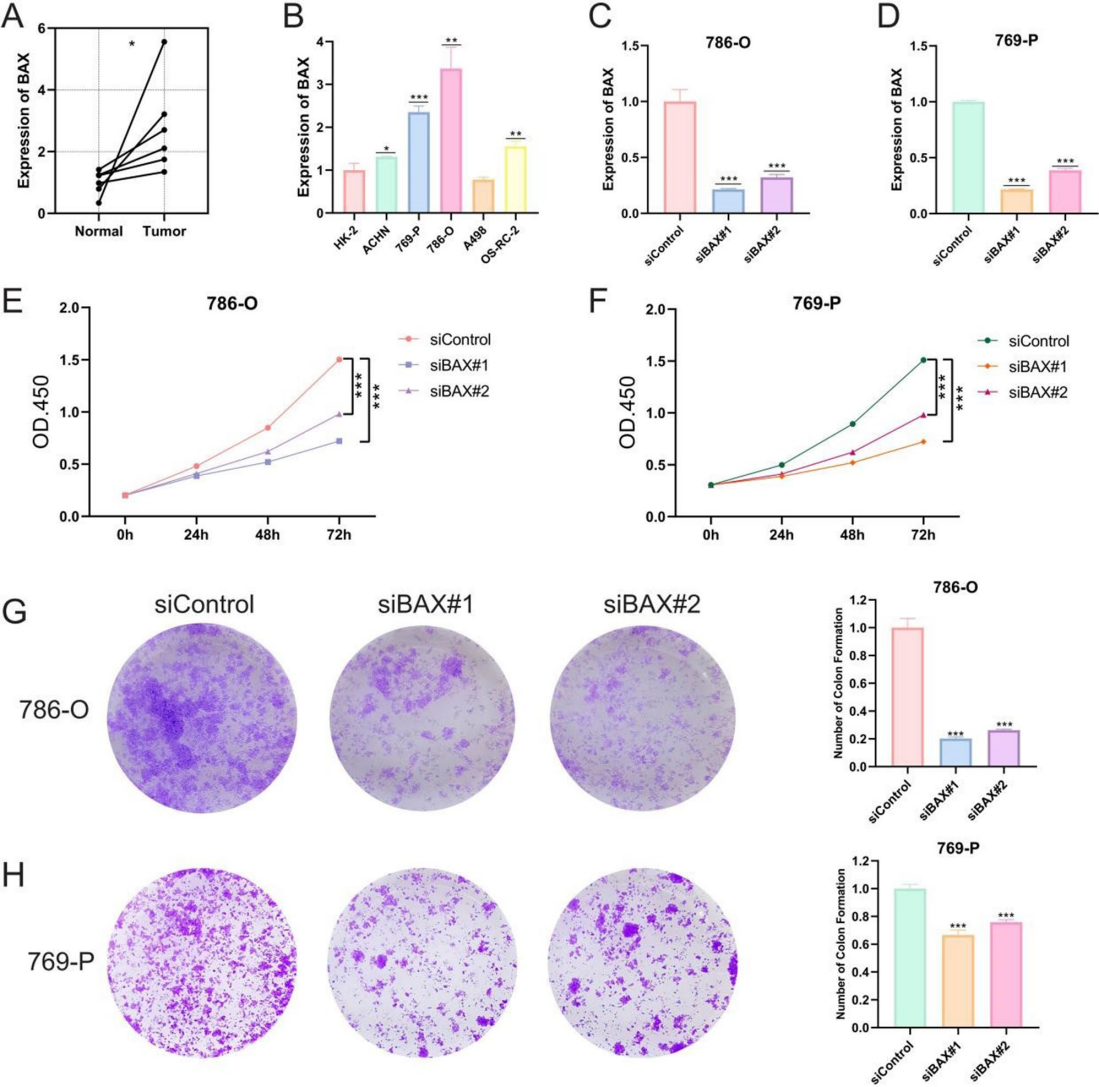
BAX expression and functional impact in ccrcc cells. (**A**) Expression difference of BAX in adjacent normal tissue and tumor tissue was analyzed in pairs. (**B**) Comparison of BAX mRNA levels in normal tubular epithelial cells (HK-2) and ccRCC cell lines. (**C**, **D**) qRT-PCR to validate BAX knockdown efficiencies in 786-O and 769-P cells (siBAX vs. siControl). (**E**, **F**) CCK-8 assay demonstrated that BAX silencing significantly inhibited the proliferation of 786-O and 769-P cells. (**G**, **H**) Colony formation assays showed that BAX knockdown decreased the clonogenesis ability of cells, and quantitative analysis was shown on the right

**Fig. 8:**
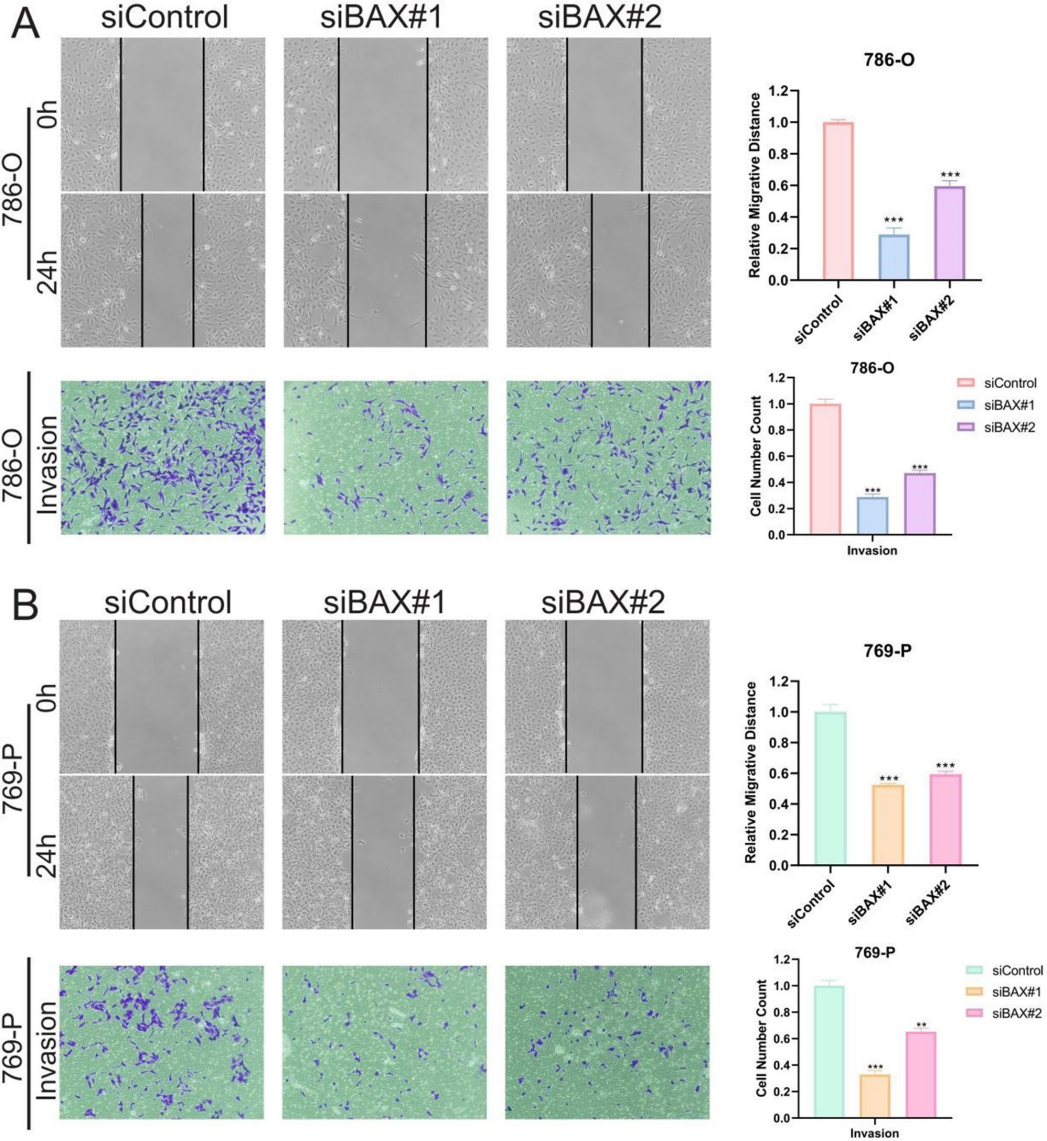
Migration and invasion assays following bax knockdown. (**A**) The migration and invasion changes of 786-O cells after BAX knockout were shown in wound healing (top) and Transwell matrix gel invasion (bottom) experiments. The migration distance and the number of invaded cells were quantized in the histogram. (**B**) Parallel experiments in 769-P cells demonstrated that BAX silencing significantly inhibited migration and invasion

Comparative analysis showed that BAX expression was significantly elevated in ccRCC tumor tissues compared with paired adjacent normal tissues (Fig. 7A), which was further confirmed in multiple ccRCC cell lines relative to normal renal epithelial cells (Fig. 7B). Efficient silencing of BAX was achieved in 786-O and 769-P cell lines via siRNA transfection (Fig. 7C-D). Functional assays revealed that BAX knockdown markedly suppressed tumor cell proliferation, as evidenced by reduced cell viability in the CCK-8 assay over time (Fig. 7E-F). Colony formation assays demonstrated that silencing BAX significantly impaired the clonogenic capacity of both cell lines (Fig. 7G-H). Furthermore, wound healing assays showed diminished migratory ability following BAX knockdown (Fig. 8A), and Transwell invasion assays confirmed that BAX silencing attenuated the invasive potential of ccRCC cells (Fig. 8B). Collectively, these results identify BAX as a potential oncogenic factor in ccRCC, whose elevated expression promotes tumor progression by enhancing proliferation, migration, invasion, and clonogenicity.

## Discussion

ccRCC has significant metabolic reprogramming and immune microenvironment remodeling characteristics, which are key factors driving tumor aggressiveness, treatment resistance and poor prognosis [18, 19]. Recently, ammonia-related cell death has been identified as a novel regulatory cell death mode, which is characterized by ammonia metabolism disorder, oxidative stress induction and mitochondrial function impairment. Although the exact role of ammonia-related cell death in the pathogenesis of ccRCC has not been fully elucidated, available evidence suggests that it may participate in the disease process by disrupting cell homeostasis, modulating immune responses, and influencing tumor biological behavior.

Importantly, the rationale for focusing on ammonia-related cell death in ccRCC is strongly supported by the unique metabolic characteristics of renal physiology and pathology. The kidney, especially the proximal tubules, plays a central role in systemic ammonia metabolism, including glutamine catabolism, acid-base homeostasis, and nitrogen excretion. Notably, ccRCC originates predominantly from proximal tubular epithelial cells, which are metabolically active and inherently exposed to ammonia flux due to their physiological role in ammoniagenesis. In the context of tumorigenesis, ccRCC cells frequently exhibit altered glutamine metabolism, defective urea cycle enzyme expression, and mitochondrial dysfunction, all of which contribute to intracellular ammonia accumulation and disruption of nitrogen homeostasis [18]. These metabolic alterations not only promote oxidative stress and mitochondrial damage but may also act as key triggers for non-canonical cell death pathways, including ammonia-related cell death [20]. Thus, ccRCC represents a biologically plausible and clinically relevant model for studying ammonia dysregulation and its pathological consequences. By targeting this metabolic vulnerability, our study sheds light on the potential of ammonia-related cell death mechanisms to serve as prognostic indicators and therapeutic targets specifically tailored to the metabolic phenotype of ccRCC.

This study establishes the dual value of ammonia-related cell deathas a prognostic biomarker and therapeutic target for ccRCC. By integrating bioinformatic analysis and experimental validation, we developed and rigorously validated a robust prognostic AS model that demonstrated good predictive power in multiple independent cohorts. Patients with high AS had significantly worse clinical outcomes, confirming the clinical value of this model and highlighting the critical role of ammonia metabolism in the progression of ccRCC. Our understanding of cancer pathogenesis is expanding with the deepening understanding of regulatory cell death pathways such as iron death, necrotic apoptosis, pyrodeath, and newly discovered copper death in tumor biology.This study further expands this cognitive paradigm by demonstrating that ammonia-related cell death (characterized by ammonia accumulation and secondary oxidative damage) is another important mechanism in the development of ccRCC. Elevated ammonia levels are known to cause mitochondrial dysfunction, oxidative stress, and disruption of cell homeostasis, which together promote malignant transformation and tumor progression [21, 22]. Supporting this view, our study found that high AS scores were significantly associated with: (1) increased tumor mutation load, (2) enhanced genomic instability, and (3) accumulation of immunosuppressive cell populations. These associations strongly suggest that the disturbance of ammonia metabolism promotes the formation of an immunosuppressive tumor microenvironment conducive to the progression of ccRCC.

It is worth noting that this study established BAX as a key regulatory gene of ammonia metabolism for the first time, and its expression level is significantly correlated with the clinical features and prognosis of ccRCC [23]. As a pro-apoptotic member of the Bcl-2 protein family, BAX is usually involved in apoptosis induction and maintenance of tissue homeostasis [24–26]. However, this study found that BAX was consistently highly expressed in ccRCC tissues, and its expression intensity was significantly positively correlated with advanced tumor stage, metastasis progression and poor prognosis, suggesting that BAX may play a key role in promoting cancer in the biological behavior of ccRCC. Functional experiments further confirmed that BAX gene silencing can significantly inhibit the proliferation, clonal formation, migration and invasion of tumor cells in vitro. This finding is highly consistent with the hypothesis that BAX promotes cancer in the context of ammonia metabolism imbalance. Interestingly, although BAX is classically recognized as a pro-apoptotic protein that promotes mitochondrial outer membrane permeabilization and caspase activation, our findings reveal a paradoxical association between high BAX expression and poor prognosis in ccRCC. This suggests that BAX may undergo functional reprogramming under the influence of ammonia metabolic dysregulation and the immunosuppressive tumor microenvironment. Several studies have indicated that the function of BAX is highly context-dependent and may be modulated by its subcellular localization, post-translational modifications, and interacting partners. For example, cytoplasmic BAX has been shown to exert non-apoptotic roles unrelated to mitochondrial permeabilization [27]. Additionally, oxidative and metabolic stress, including elevated ammonia levels, may lead to altered conformational states of BAX or influence its interactions with other cellular proteins [4, 28]. Furthermore, BAX has been implicated in modulating mitochondrial dynamics and calcium signaling independent of apoptosis [29], suggesting that in a high-ammonia environment, such as that seen in ccRCC, BAX may acquire a pro-tumorigenic function by supporting metabolic adaptation or immune evasion mechanisms [30]. These possibilities highlight the need for further investigation into the non-canonical roles of BAX in tumor biology, particularly within metabolically reprogrammed and immunosuppressive niches. Immunomicroenvironment analysis revealed significant enrichment of immunosuppressive cell populations, including MDSCs, Tregs, and tumor-associated macrophages, in tumors with high AS. Along with the upregulation of immune checkpoint molecules, it is suggested that the disturbance of ammonia metabolism may promote tumor immune escape through the formation of immunosuppressive microenvironment, and then lead to immunotherapy resistance [31]. Clinical validation based on the IMvigor210 cohort confirmed that objective response rates and survival were significantly reduced in patients with high AS receiving immune checkpoint blocking therapy, suggesting that AS can be used as a reliable biomarker to predict immunotherapy resistance. Combining AS assessment with traditional clinical parameters is expected to improve the precision of individualized immunotherapy strategies. Mechanistically, the immunosuppressive phenotype observed in the high-AS group may result from the interplay between ammonia-induced metabolic stress and key immune regulatory pathways. Elevated intracellular ammonia can promote oxidative stress, which in turn activates NF-κB and STAT3, both of which are known to induce PD-L1 expression and facilitate immune escape. Concurrently, BAX-mediated mitochondrial dysfunction may lead to release of damage-associated molecular patterns (DAMPs), which reprogram immune cells—particularly tumor-associated macrophages and Tregs—toward immunosuppressive phenotypes. Moreover, TGF-β signaling, enriched in high-AS tumors, is a well-established driver of T cell exhaustion, M2 macrophage polarization, and stromal activation. These pathways may be further enhanced by ammonia-related shifts in metabolic intermediates (e.g., α-ketoglutarate), which influence epigenetic reprogramming and immune checkpoint upregulation. Together, these findings suggest that the AS score not only reflects a prognostic signature, but also captures a complex immune-metabolic suppression axis driven by tumor-intrinsic ammonia dysregulation.

Interestingly, recent studies have shown that BAX may not act solely through classical apoptotic mechanisms but could also interact with other regulated cell death (RCD) pathways. While BAX is canonically associated with mitochondrial apoptosis, it may serve as a convergence node with other RCD forms such as ferroptosis and cuproptosis, particularly under metabolic stress like ammonia accumulation. For instance, BAX-mediated mitochondrial permeabilization has been linked to enhanced lipid peroxidation in ferroptosis [23, 32], and the mitochondrial metabolic context of cuproptosis may engage BAX through proteotoxic or redox-sensitive mechanisms [33]. These observations raise the possibility that BAX may function as an integrative effector influenced by post-translational modifications or non-canonical interactors under the tumor’s altered metabolic landscape [34]. Further mechanistic studies are warranted to investigate whether BAX-mediated ammonia-related cell death is modulated by or intersects with other cell death programs, which could reveal novel combinatorial therapeutic strategies targeting multi-modal vulnerabilities in ccRCC.

Despite the strengths of our multi-omics integration and experimental validation, several limitations should be acknowledged. First, although our model was validated across multiple public cohorts, the absence of prospective clinical validation limits its immediate translational applicability. Future studies will include multi-center cohorts and prospective patient samples to confirm the prognostic utility of the AS model. Second, while we identified BAX as a key ammonia-related regulator, the precise molecular pathways linking BAX to ammonia stress and immune suppression remain incompletely defined. Third, the mechanistic implications of ammonia metabolism on cell death heterogeneity, especially its intersection with other regulated cell death forms (e.g., ferroptosis, pyroptosis), require further exploration. We plan to conduct metabolomic and proteomic profiling under ammonia-loading conditions to uncover new metabolic checkpoints and their regulatory networks. By addressing these limitations through rigorous experimental design, we aim to enhance the translational and mechanistic impact of our findings in future work.

## Conclusion

In summary, this study is the first to systematically elucidate the prognostic value and therapeutic potential of ammonia-related cell death in ccRCC. We constructed and validated a prognostic model based on ammonia-related cell death that reliably predicted clinical outcomes and immunotherapy responses. Crucially, it was found that BAX gene, as the core effector molecule in the regulation of ammonia-related cell death, has the dual value of tumor promoting biomarker and therapeutic target. These findings not only deepen the understanding of the pathogenesis of ccRCC from the perspective of ammonia-related cell death, but also lay a theoretical foundation for the precise treatment strategy targeting the ammonia-related cell death pathway.

## Electronic supplementary material

Below is the link to the electronic supplementary material.

Supplementary Material 1

## Data Availability

No datasets were generated or analysed during the current study.
