# Supplementary material for: BAX-mediated ammonia-driven cell death: a novel prognostic and therapeutic target in clear cell renal cell carcinoma
Source: Hum Genomics. 2025 May 17;19:57. doi: 10.1186/s40246-025-00764-3 (PMC12085854; doi:10.1186/s40246-025-00764-3)
Supplement: Supplementary file 1 — Supplementary Material 1 [file 40246_2025_764_MOESM1_ESM.docx]

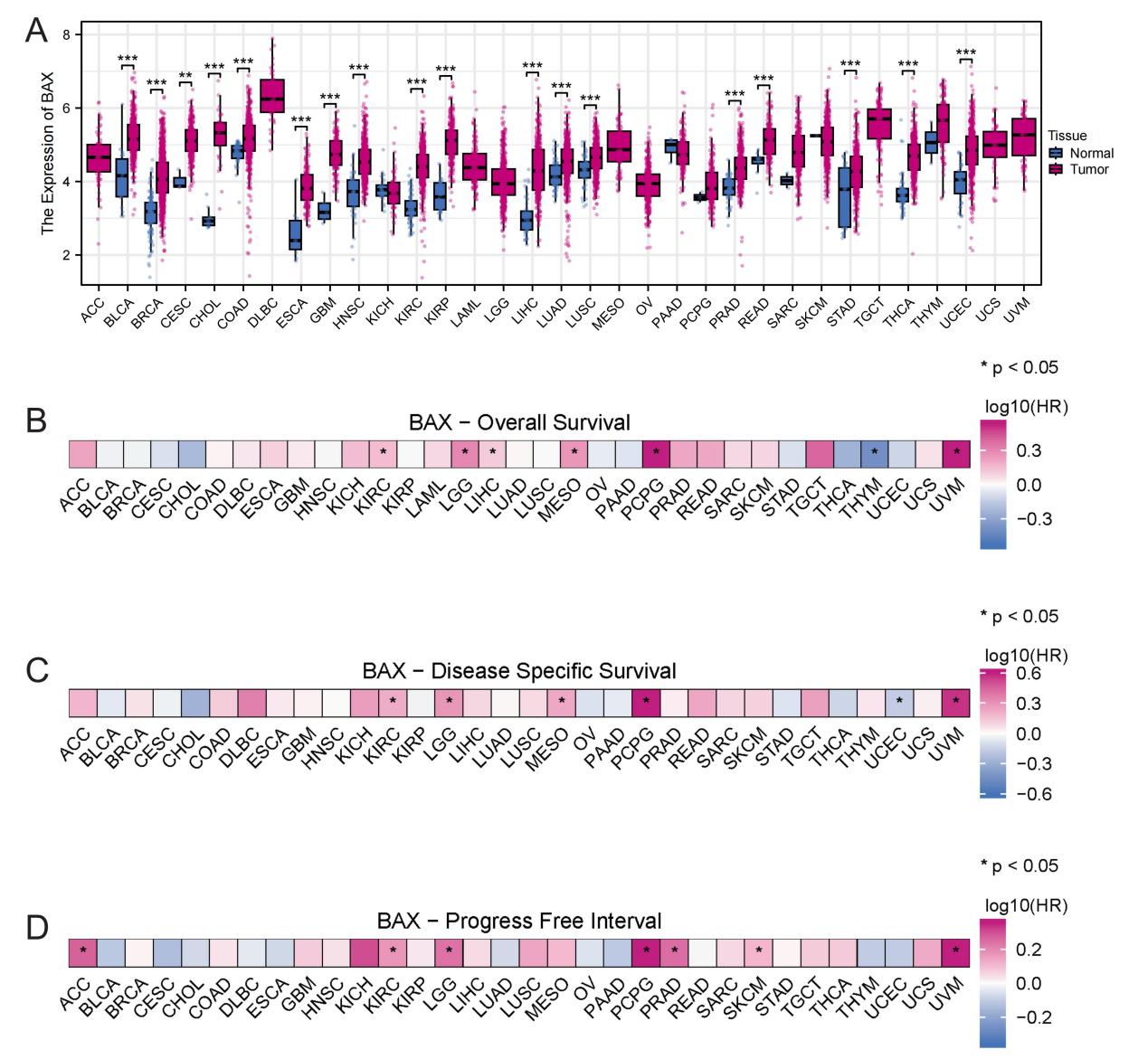


**Figure S1. Pan-cancer Expression and Prognostic Relevance of BAX across TCGA Cancer Types.** (A) Boxplot comparing BAX expression between tumor tissues (pink) and normal tissues (blue) across 33 cancer types. (B) Heatmap showing the association between BAX expression and overall survival (OS); (C) Disease-specific survival (DSS); (D) Progression-free interval (PFI). Significance: *P < 0.05, **P < 0.01, ***P < 0.001.
